# Quality of Life and Mental Health of Chinese Sexual and Gender Minority Women and Cisgender Heterosexual Women: Cross-sectional Survey and Mediation Analysis

**DOI:** 10.2196/42203

**Published:** 2023-02-22

**Authors:** Chanchan Wu, Pui Hing Chau, Edmond Pui Hang Choi

**Affiliations:** 1 School of Nursing The University of Hong Kong Hong Kong China (Hong Kong)

**Keywords:** quality of life, depression, anxiety, self-esteem, sexual and gender minority women, mental health, mediation analysis, China, minority, women, survey, social

## Abstract

**Background:**

Health-related research on sexual minority populations in China is lacking, and research on sexual and gender minority women (SGMW, including transgender women and persons of other gender identities assigned female at birth of all sexual orientations, and cisgender women with nonheterosexual orientations) is even less. Currently, there are limited surveys related to mental health in Chinese SGMW, but there are no studies on their quality of life (QOL), no studies comparing the QOL of SGMW with that of cisgender heterosexual women (CHW), and no studies on the relationship between sexual identity and the QOL as well as associated mental health variables.

**Objective:**

This study aims to evaluate the QOL and mental health in a diverse sample of Chinese women and make comparisons between SGMW and CHW and then investigate the relationship between sexual identity and the QOL through the role of mental health.

**Methods:**

A cross-sectional online survey was conducted from July to September 2021. All participants completed a structured questionnaire containing the World Health Organization Quality of Life–abbreviated short version (WHOQOL-BREF), the 9-item Patient Health Questionnaire (PHQ-9), the 7-item Generalized Anxiety Disorder scale (GAD-7), and the Rosenberg Self-Esteem Scale (RSES).

**Results:**

In total, 509 women aged 18-56 years were recruited, including 250 (49.1%) CHW and 259 (50.9%) SGMW. Independent *t* tests showed that the SGMW reported significantly lower levels of QOL, higher levels of depression and anxiety symptoms, and lower self-esteem than the CHW. Pearson correlations showed that every domain and the overall QOL were positively associated with mental health variables, with moderate-to-strong correlations (r range 0.42-0.75, *P*<.001). Multiple linear regressions found that participants belonging to the SGMW group, current smokers, and women with no steady partner were associated with a worse overall QOL. The mediation analysis found that depression, anxiety, and self-esteem significantly completely mediated the relationship between sexual identity and physical, social, and environment domains of the QOL, while the relationship between sexual identity and the overall QOL and psychological QOL was partially mediated by depression and self-esteem.

**Conclusions:**

The SGMW had poorer levels of QOL and a worse mental health status than the CHW. The study ﬁndings afﬁrm the importance of assessing mental health and highlight the need to design targeted health improvement programs for the SGMW population, who may be at higher risk of a poor QOL and mental health.

## Introduction

Public health surveillance systems, to date, have focused on cisgender heterosexual (CH) individuals, whose gender identity is consistent with their sex assigned at birth and who are sexually or romantically attracted to people of the opposite sex, while people with other gender and sexual orientation identities have historically been less visible [1]. In general, all non-CH individuals could be characterized as sexual and gender minority (SGM) populations. Specifically, gender minorities include but are not limited to people who are transgender (whose gender identity and sex assigned at birth do not correspond), gender queer (whose gender identity falls outside the traditional gender), gender fluid (whose gender identity is not fixed), gender questioning (who are unsure about or are exploring their gender identity), and other nonbinary genders outside of the traditional gender binary of male and female. Similarly, sexual orientation minorities include but are not limited to homosexual (gay and lesbian, people who are primarily sexually or romantically attracted to people of the same sex or gender as themselves), bisexual (attraction to multiple genders), pansexual (attraction to all genders/regardless of gender), and asexual (who experience little or no sexual attraction to others) or other nonheterosexual orientations.

A growing body of research has explored health disparities among people of different sexual orientations, and sexual minority populations have consistently been found to report worse health outcomes than their CH peers, especially in the area of mental health [2-9]. Specifically, studies investigating mental health and its relationship with sexual orientations have shown that sexual minority populations are more likely to experience mental health problems than CH individuals, with depression and anxiety being the most commonly diagnosed mental health disorders [3,10-16]. In a comprehensive meta-analysis of large samples, sexual minority populations were twice as likely to suffer from depression and anxiety as CH individuals [17]. It is worth noting that from a gender perspective, mental disorders are more commonly diagnosed in women than in men globally [18,19], so relevant research on women is crucial. Even in the field of research on SGM populations, more attention has been paid to males than females [20]; therefore, sexual and gender minority women (SGMW, including transgender women and persons of other gender identities assigned female at birth of all sexual orientations, and cisgender women with nonheterosexual orientations) need more public health attention.

As emphasized by the World Health Organization (WHO), mental health is “not just the absence of mental disorder” but “a state of well-being in which individuals realize their own potential and can cope with normal stresses of life” [19]. In other words, a healthy mental state is not only the absence of depression, anxiety, or other symptoms but also the presence of positive components of well-being. Self-esteem, literally deﬁned as the degree to which individuals believe themselves to be valuable or adequate [21], is one such essential component and strongly related to happiness [22]. Similar to gender differences in mental disorders, a meta-analysis of self-esteem also revealed that women report lower levels of self-esteem than men [23]. Although high levels of self-esteem are associated with positive self-attitudes and are protective against adverse mental health outcomes [24], low levels of self-esteem, more commonly reported by people belonging to sexual minority populations [25], increase the likelihood of mental health difficulties.

In addition to mental health, a poor quality of life (QOL) has been shown to be positively associated with adverse mental health outcomes [26]. The QOL is widely used to present individuals’ perceptions of their physical and psychological health, social relationships, and environment [27]. However, there is compelling evidence that sexual minority populations are more likely to report a poorer QOL than CH individuals [28], especially psychological QOL [29]. To promote the overall health of SGM populations, it is not sufficient to only examine differences in the incidence of mental disorders and levels of the QOL without understanding the in-depth relationships and mechanisms among them. A recent review examining mediators of the depression disparities between sexual minority and CH populations suggested that the psychological process may also play a role, but this has been understudied to date [30], and more mediation analyses are needed.

Health-related research on SGMW is growing slowly worldwide [31-33], but there is less research on Chinese SGMW. According to published data from Western countries, sexual minority women experience a higher incidence of mental health disorders, including anxiety and depression, and also generally report a worse QOL than cisgender heterosexual women (CHW) [34,35]. A recent scoping review summarizing the holistic health of homosexual and bisexual Chinese over the past 20 years showed that the academic attention given to the male population is much higher than that given to female groups (96.46% vs 1.32%, respectively) [20]. Although there are limited surveys investigating the mental health of SGMW in China, results regarding depression and anxiety are less consistent among women from different countries with varied sexual identities [13,36-38]. Furthermore, there is currently no research on the QOL of Chinese SGMW and the relationship between sexual identity and the QOL, highlighting the need to research the health of women, especially SGMW in China.

The associations between sexual identity and the QOL as well as mental health vary across studies conducted in different countries, and there are currently no studies aimed at explaining these relationships among Chinese SGM populations, let alone SGMW. Therefore, this study aims to first examine the QOL and mental health of Chinese adult women of different sexual identities, with mental health variables including depression and anxiety as well as self-esteem, and then investigate the relationships between sexual identity and the QOL through the role of mental health variables in the Chinese context. Our main hypotheses were as follows: (1) SGMW in China may report lower levels of the QOL and more mental health disorders compared to CHW, (2) a better mental health status may positively be associated with higher levels of the QOL, and (3) mental health variables mediate the effect of sexual identity on the QOL.

## Methods

### Participants and Procedures

This was a cross-sectional study conducted online in China. Given the relative sensitivity and invisibility of our study population under the influence of traditional Chinese culture, multiple recruitment methods were used, including (1) convenient recruitment via 4 popular nongovernmental organizations (NGOs) serving female or SGM populations in mainland China (TrueSelf, r&B bisexual community, Period Pride China, and the Wuhan LGBT Center); (2) respondent-driven recruitment, where respondents were encouraged to help recruit potential peers through their network of connections [39,40]; and (3) social platform recruitment by releasing the study poster to some popular online communities.

Eligible participants included Chinese women who were (1) at least 18 years old; (2) self-identiﬁed as “female,” either cisgender or transgender women or gender nonbinary individuals whose sex assigned at birth was female; and (3) able to read and understand Mandarin. Data were collected using an online survey platform (Wenjuanxing), and data collection was initiated after informed consent was obtained.

### Ethical Considerations

Ethical approval was obtained from the Human Research Ethics Committee (HREC) of the University of Hong Kong (reference no. EA210325). All participants provided informed consent.

### Study Instruments

Participants who consented to join the study were asked to ﬁll out all the following questionnaires in Mandarin.

#### The 9-Item Patient Health Questionnaire

The 9-item Patient Health Questionnaire (PHQ-9) is a depression module that measures the presence and severity of depression symptoms over the past 2 weeks according to the *Diagnostic and Statistical Manual of Mental Disorders IV* (DSM-IV) criteria for assessing symptoms of depression [41]. The PHQ-9 is a self-report questionnaire, and each item ranges from 0 (not at all) to 3 (nearly every day), with a summed score ranging from 0 to 27. The Chinese version of the PHQ-9 has been previously evaluated for reliability and validity, showing that it is a valid and efﬁcient tool for screening depression [42]. This study used this Chinese version of the PHQ-9, and the Cronbach α of the scale among the study sample was .91.

#### The 7-Item Generalized Anxiety Disorder Scale

The 7-item Generalized Anxiety Disorder scale (GAD-7) is a self-reported screening tool to identify anxiety symptoms in the past 2 weeks. Each of the 7 items is scored from 0 (not at all) to 3 (nearly every day), and the GAD-7 scale score ranges from 0 to 21 [43]. The Chinese version of GAD-7 has been previously validated and is commonly used [44,45]. This study used this Chinese version, and the Cronbach α of the scale among the study sample was .94.

#### The Rosenberg Self-Esteem Scale

The Rosenberg Self-Esteem Scale (RSES) is a 10-item self-report scale evaluating an individual’s self-esteem [21]. Each item is answered using a 4-point Likert scale ranging from 1 (strongly agree) to 4 (strongly disagree), while half items are reverse-scored ranging from 1 (strongly disagree) to 4 (strongly agree). The Chinese version of the questionnaire has been previously validated [46-49], and this study used this Chinese version to measure participants’ self-esteem. All scores of the 10 items are summed as a global score on a continuous scale, and higher scores indicate lower overall self-esteem. In this study, the Cronbach α of the scale was .77.

#### The World Health Organization Quality of Life–Abbreviated Short Version

The World Health Organization Quality of Life–abbreviated short version (WHOQOL) is a QOL assessment tool developed by the WHOQOL Group and includes 100 items on a 5-point scale [27]. Based on that, an abbreviated short version of the WHOQOL instrument (WHOQOL-BREF) has been developed and is widely used in assessing the QOL of the public [50,51]. The WHOQOL-BREF contains 26 items in total, including 2 (8%) overall items and 24 (92%) facet items on 4 domains: physical (7, 29%, items), psychological (6, 25%, items), social relationships (3, 13%, items), and environment (8, 33%, items). Regarding the scoring, there are 3 negatively phrased items (items 3, 4, and 26) that should be reversed before calculating the scores. The mean score of items within each domain is used to calculate the domain score, and each domain score can be transformed into a score ranging from 4 to 20, with higher scores representing a better QOL [27,51,52]. The Chinese version of the WHOQOL-BREF has been previously validated [51] and was used in this study. In this study, the Cronbach α coefficient of the total scale was .93 and of the 4 subscales was .79, .88, .68, and .83, respectively.

#### Sociodemographic Information

All participants completed a set of sociodemographic items, including their basic information (age, employment, race/ethnicity, education level, income, relationship status) and their sexual identity (gender identity and sexual orientation identity).

### Sample Size Justiﬁcation

The sample size calculation was performed using G*Power 3.1 [53]. To detect a statistically signiﬁcant difference between CHW and SGMW using the independent samples *t* test with 80% power, 5% level of significance (2-tailed), and a Cohen *d* effect size of 0.3, a minimum sample size of 352 participants was needed.

### Statistical Analysis

Descriptive statistics (counts and percentages, means, and SDs) were used to illustrate participants’ demographic characteristics, QOL, and mental health outcomes.

#### Comparisons Between SGMW and CHW

To compare the scores of mental health variables (PHQ-9, GAD-7, RSES) and the QOL (WHOQOL-BREF) between SGMW and CHW, independent *t* tests were conducted and the Cohen *d* effect size was also calculated, with cut-offs of 0.2, 0.5, and 0.8 for small, medium, and large effect sizes, respectively [54]. To compare the percentages of participants’ demographic characteristics, chi-square tests were conducted. Pearson correlations were used to identify the correlations between mental health variables and the QOL, and the correlations were defined as strong (≥0.5), moderate (≥0.3 and <0.5), or weak (<0.3). One-way ANOVA was performed to compare the mean scores in 5 sexual orientation groups. Next, multiple linear regression analyses were performed to identify factors associating mental health and the QOL separately.

#### Parallel Mediation Analysis

To examine mediating effects, we used a bootstrapping technique using the PROCESS macro [55], and parallel mediation models were assessed. We implemented 5000 bootstrap samples for the percentile bootstrap CI, and a 95% confidence level was used. We proposed the effects of sexual identity (independent X: sexual minority vs majority) through 3 mediating mental health variables (parallel mediators: M1, depression; M2, anxiety; and M3, self-esteem) on the QOL (dependent Y: physical QOL, psychological QOL, social relationship QOL, environment QOL, and overall QOL). The existing literature has suggested that many sociodemographic factors are associated with the QOL [55-57], so age, income, alcohol use, smoking status, and relationship status were included as covariates to control for their potential effects in the mediation analysis.

**Figure 1:**
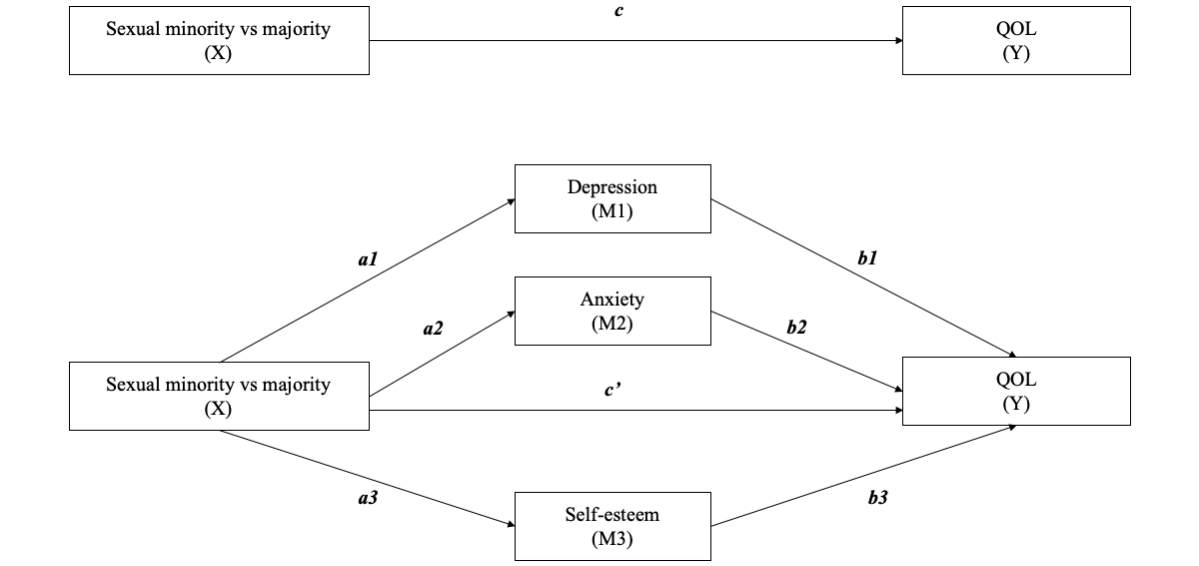
Proposed parallel mediation model; a: effect of the independent variable (X) on mental health (M1-M3), b: effect of mental health on QOL (Y), c: total effect of X on Y, c’: direct effect of X on Y. M: mediator; QOL: quality of life.

As shown in Figure 1, “a” is the effect of the independent variable (X) on mental health (mediators M1-M3), “b” is the effect of mental health (M1-M3) on the QOL (Y), “c” is the total effect of X on Y, and c’ is the direct effect of X on Y. An indirect effect was considered signiﬁcant if the CI did not contain 0. If the indirect effect is significant, it means that there is a mediation effect. Meanwhile, if the direct effect disappears, then there is complete mediation, whereas if the direct effect remains, then there is partial mediation [58]. Thus, we expected that sexual minority status would be positively associated with mental disorders (higher levels of depression and anxiety but lower levels of self-esteem) and, in turn, be negatively associated with the QOL.

Data were analyzed using IBM Statistical Package for the Social Sciences (SPSS) version 27.0, and the PROCESS macro version 4.1 for SPSS was used to conduct the mediation analysis [55]. All signiﬁcance tests were 2-tailed, and ﬁndings with *P*<.05 were considered statistically signiﬁcant.

## Results

### Demographic Characteristics of Study Participants

Of the 524 questionnaires collected between July and September 2021, 15 (2.9%) were unqualified since they were cisgender male (9/15, 60%) or less than 18 years old (4/15, 27%) or invalid data with all same answers (2/15, 13%), resulting in a total of 509 (97.1%) adult women enrolled in the study with no missing data. Specifically, 250 (49.1%) were CHW and categorized as the CHW group, while 259 (50.9%) were either sexual orientation or gender minority women and categorized as the SGMW group. The sexual orientation and gender identity of participants are shown in Table 1.

The mean age of the overall sample was 25.57 (SD 5.77) years, ranging from 18 to 56 years (Table 2). The mean age of the CHW was 26.75 (SD 6.31) years and was 24.43 (SD 4.94) years for the SGMW. Most participants were Han people (n=466, 91.6%) in the age group of 21-30 years (n=347, 68.2%) and had college or bachelor’s degrees (n=284, 55.8%), and around two-fifths were full-time students (n=222, 43.6%). About half of the women were single, and half had a steady partner (n=258, 50.7%, vs n=251, 49.3%), and a total of 248 (48.7%) women had full-time jobs. One-third of the women (n=178, 35.0%) never had a drink before, and the majority (n=428, 84.1%) had never smoked or had already quit smoking. Notably, 9 (1.8%) participants had a history of drug use.

**Table 1:** Frequency distribution of participants by gender and sexual orientation identity (N=509).

Gender identity	Sexual orientation identity, n (%)					
	Homosexual	Heterosexual	Bisexual	Pansexual	Others^a^	Total
Cisgender women	51 (10.0)	250 (49.1)^b^	74 (14.5)	33 (6.5)	28 (5.5)	436 (85.7)
Transgender women	8 (1.6)	1 (0.2)	1 (0.2)	1 (0.2)	2 (0.4)	13 (2.6)
Gender queer	10 (2.0)	1 (0.2)	4 (0.8)	12 (2.4)	1 (0.2)	28 (5.5)
Gender fluid	0	0	4 (0.8)	2 (0.4)	1 (0.2)	7 (1.4)
Other gender nonbinary^c^	2 (0.4)	7 (1.4)	5 (1.0)	4 (0.8)	7 (1.4)	25 (4.9)
Total	71 (13.9)	259 (50.9)	88 (17.3)	52 (10.2)	39 (7.7)	509 (100.0)

^a^Other minority sexual orientation identities, including asexual, gynephilia/femininity, and all other nonheterosexual orientations.

^b^The cisgender heterosexual women (CHW) group in this study comprised 250 (49.1%) women. Correspondingly, the other 259 (50.9%) sexual orientation and gender minority women were categorized as the sexual and gender minority women (SGMW) group.

^c^This group included gender questioning (someone who is unsure about or is exploring their gender identity), no gender, and other nonbinary genders that are noncisgender and beyond social norms.

**Table 2:** Characteristics of the study sample with comparisons.

Characteristics		Overall (N=509)	SGMW^a^ (n=259, 50.1%)	CHW^b^ (n=250, 49.1%)
**Age (years), n (%)**				
	18-20	94 (18.5)	61 (23.6)	33 (13.2)
	21-30	347 (68.2)	171 (66.0)	176 (70.4)
	31-40	56 (11.0)	25 (9.7)	31 (12.4)
	41-56	12 (2.4)	2 (0.8)	10 (4.0)
Mean age, mean (SD); *P*<.001^c^		25.57 (5.77)	24.43 (4.94)	26.75 (6.31)
**Number of friends, n (%)**				
	0	144 (28.3)	48 (33.3)	96 (66.7)
	1-5	154 (30.3)	96 (62.3)	58 (37.7)
	6-10	74 (14.5)	50 (67.6)	24 (32.4)
	11-20	43 (8.4)	28 (65.1)	15 (34.9)
	21-50	40 (7.9)	20 (50.0)	20 (50.0)
	51-100	27 (5.3)	12 (44.4)	15 (55.6)
	>100	27 (5.3)	5 (18.5)	22 (81.5)
Mean numbers of friends around, mean (SD); *P*=.005		33.44 (108.28)	20.14 (71.40)	47.22 (135.14)
**Education, n (%); *P*<.001**				
	High school and below	33 (6.5)	21 (8.1)	12 (4.8)
	College/bachelor	284 (55.8)	167 (64.5)	117 (46.8)
	Graduate degree and above	192 (37.7)	71 (27.4)	121 (48.4)
**Ethnic, n (%); *P*=.12**				
	Han people	466 (91.6)	242 (93.4)	224 (89.6)
	Others (Muslim, Zhuang people, etc)	43 (8.4)	17 (6.6)	26 (10.4)
**Employment, n (%); *P*<.001**				
	Unemployed	39 (7.7)	29 (11.2)	10 (4.0)
	Full-time student	222 (43.6)	118 (45.6)	104 (41.6)
	Organization officer	42 (8.3)	23 (8.9)	19 (7.6)
	Professional/technical personnel	103 (20.2)	29 (11.2)	74 (29.6)
	Administrative personnel	17 (3.3)	7 (2.7)	10 (4.0)
	Service personnel	33 (6.5)	19 (7.3)	14 (5.6)
	Others (famers/freelancers)	53 (10.4)	34 (13.1)	19 (7.6)
**Monthly income (CNY/US $^d^), n (%);*P*<.001**				
	≤CNY 5000.00/US $743.89	282 (55.4)	162 (62.5)	120 (48.0)
	>CNY 5000.00/US $743.89	227 (44.6)	97 (37.5)	130 (52.0)
**Relationship, n (%); *P*<.001**				
	Have a steady partner	251 (49.3)	115 (44.4)	136 (54.4)
	Have no steady partner(s)	258 (50.7)	144 (55.6)	114 (45.6)
**Alcohol use, n (%); *P*<.001**				
	Never drank before	178 (35.0)	68 (26.3)	110 (44.0)
	Current alcohol user	331 (65.0)	191 (73.7)	140 (56.0)
**Drug use, n (%); *P*=.10**				
	Never used before	500 (98.2)	252 (97.3)	248 (99.2)
	Have used drug before	9 (1.8)	7 (2.7)	2 (0.8)
**Smoking, n (%); *P*<.001**				
	Never smoked or have quit smoking	428 (84.1)	188 (72.6)	240 (96.0)
	Current smoker	81 (15.9)	71 (27.4)	10 (4.0)

^a^SGMW: sexual and gender minority women. The percentages for this column were all calculated with 259 SGMW as the denominator.

^b^CHW: cisgender heterosexual women. The percentages for this column were all calculated with 250 CHW as the denominator.

^c^All *P* values were reported by conducting comparisons performing independent *t* tests or chi-square tests.

^d^CNY 1=US $0.15.

### QOL and Mental Health Among Study Participants With Comparisons

Table 3 shows the mean scores of the QOL (as measured by the WHOQOL-BREF) and mental health outcomes (as measured by the PHQ-9, GAD-7, and RSES) for our study participants. The mean QOL scores of each domain in the overall sample were ranked as follows: physical QOL (mean 14.05, SD 2.71), psychological QOL (mean 13.74, SD 3.13), environment QOL (mean 13.74, SD 2.68), and social relationship QOL (mean 13.62, SD 3.07). The mean scores of mental health variables in the overall sample were 7.85 (SD 5.92) for depression, 6.00 (SD 5.23) for anxiety, and 28.86 (SD 4.66) for self-esteem. All comparisons indicated that compared to the CHW group, the SGMW group reported significantly lower levels of overall QOL (52.77 vs 57.61) and QOL in all domains, lower levels of self-esteem (28.04 vs 29.72), but higher levels of depression (9.16 vs 6.50) and anxiety (5.49 vs 4.77) symptoms (all *P*<.01). The comparisons among 5 different sexual orientation groups were also conducted (Multimedia Appendix 1).

**Table 3:** QOL^a^ and mental health of the study participants with comparisons.

QOL and mental health		Overall (N=509)	SGMW^b^ (n=259, 50.9%)	CHW^c^ (n=250, 49.1%)	*P* value	Cohen *d* (SGMW-CHW)
**QOL (WHOQOL-BREF^d^), mean (SD)**						
	Physical	14.05 (2.71)	13.49 (2.87)	14.63 (2.39)	<.001	–0.43
	Psychological	13.74 (3.13)	12.94 (3.35)	14.57 (2.65)	<.001	–0.54
	Social relationship	13.62 (3.07)	13.02 (3.35)	14.23 (2.61)	<.001	–0.40
	Environment	13.74 (2.68)	13.32 (2.90)	14.18 (2.35)	<.001	–0.33
	Total score	55.15 (9.90)	52.77 (10.69)	57.61 (8.34)	<.001	–0.50
**Mental health, mean (** **SD)**						
	Depression (PHQ-9^e^)	7.85 (5.92)	9.16 (6.36)	6.50 (5.11)	<.001	0.46
	Anxiety (GAD-7^f^)	6.00 (5.23)	6.93 (5.49)	5.04 (4.77)	<.001	0.37
	Self-esteem (RSES^g^)	28.86 (4.66)	28.04 (4.95)	29.72 (4.18)	<.001	–0.37

^a^QOL: quality of life.

^b^SGMW: sexual and gender minority women.

^c^CHW: cisgender heterosexual women.

^d^WHOQOL-BREF: World Health Organization Quality of Life–abbreviated short version.

^e^PHQ-9: 9-item Patient Health Questionnaire.

^f^GAD-7: 7-item Generalized Anxiety Disorder scale.

^g^RSES: Rosenberg Self-Esteem Scale.

### Correlations Between the QOL and Mental Health

Pearson correlations between the QOL and depression, anxiety, and self-esteem are displayed in Table 4. As hypothesized, both depression and anxiety symptoms were significantly negatively associated with all domains of the QOL as well as the overall QOL, with moderate-to-strong correlations (*r* range –0.42 to –0.68, all *P*<.001). Self-esteem was significantly positively associated with all domains of the QOL as well as the overall QOL, with all strong correlations (*r* range 0.52-0.75, all *P*<.001).

**Table 4:** Correlations between the QOL^a^ and mental health variables.

Mental health		QOL (WHOQOL-BREF^b^)				
		Physical	Psychological	Social relationship	Environment	Total score
**Depression (PHQ-9^c^)**						
	r value	–0.66	–0.67	–0.52	–0.47	–0.68
	*P* value	<.001	<.001	<.001	<.001	<.001
**Anxiety (GAD-7^d^)**						
	r value	–0.56	–0.55	–0.42	–0.45	–0.58
	*P* value	<.001	<.001	<.001	<.001	<.001
**Self-esteem (RSES^e^)**						
	r value	0.60	0.75	0.53	0.52	0.71
	*P* value	<.001	<.001	<.001	<.001	<.001

^a^QOL: quality of life.

^b^WHOQOL-BREF: World Health Organization Quality of Life–abbreviated short version.

^c^PHQ-9: 9-item Patient Health Questionnaire.

^d^GAD-7: 7-item Generalized Anxiety Disorder scale.

^e^RSES: Rosenberg Self-Esteem Scale.

### Regressions of the QOL and Mental Health

Tables 5-12 display the results of multiple linear regressions. The identity of being a sexual minority woman had a significant negative association with the QOL in all domains as well as the overall QOL (all *P*<.01). In the regression models of mental health variables, belonging to the SGMW group was associated with more severe depression (*P*=.002) and anxiety (*P*=.02) symptoms as well as lower self-esteem levels (*P*=.02). The current smoking status was significantly negatively associated with the overall QOL and all other domains of the QOL except social relationship QOL. Regarding the regressions of mental health variables, the current smoking status was significantly associated with higher levels of depression (*P*<.001) and anxiety (*P*=.006) and lower levels of self-esteem (*P*=.002). Furthermore, having a steady partner was found to be significantly positively associated with the overall QOL (*P*=.006), psychological QOL (*P*=.02), and social relationship QOL (*P*<.001).

**Table 5:** Regressions of sexual identity on the physical QOL^a^.

Characteristics	Physical QOL (R-square=7.9%)		
	Coefficient	*P* value	95% CI
Sexual identity (CHW^b^ vs SGMW^c^)	0.77	.003	0.27 to 1.26
Age	0.00	.93	–0.04 to 0.05
Alcohol user (yes vs no)	–0.40	.12	–0.90 to 0.10
Drug user (yes vs no)	1.00	.27	–0.79 to 2.79
Current smoker (yes vs no)	–0.84	.02	–1.53 to –0.15
Income (≥CNY 5000/US $743.89^d^ vs below)	0.52	.052	0.00 to 1.05
Have a steady partner (vs no)	0.41	.09	–0.07 to 0.89

^a^QOL: quality of life.

^b^CHW: cisgender heterosexual women.

^c^SGMW: sexual and gender minority women.

^d^CNY 1=US $0.15.

**Table 6:** Regressions of sexual identity on the psychological QOL^a^.

Characteristics	Psychological QOL (R-square=11.3%)		
	Coefficient	*P* value	95% CI
Sexual identity (CHW^b^ vs SGMW^c^)	1.13	<.001	0.56 to 1.69
Age	0.02	.48	–0.03 to 0.07
Alcohol user (yes vs no)	–0.11	.70	–0.68 to 0.46
Drug user (yes vs no)	0.63	.54	–1.40 to 2.66
Current smoker (yes vs no)	–1.38	.001	–2.16 to –0.60
Income (≥CNY 5000/US $743.89^d^ vs below)	0.43	.15	–0.16 to 1.03
Have a steady partner (vs no)	0.64	.02	0.10 to 1.19

^a^QOL: quality of life.

^b^CHW: cisgender heterosexual women.

^c^SGMW: sexual and gender minority women.

^d^CNY 1=US $0.15.

**Table 7:** Regressions of sexual identity on the social relationship QOL^a^.

Characteristics	Social relationship QOL (R-square=9.7%)		
	Coefficient	*P* value	95% CI
Sexual identity (CHW^b^ vs SGMW^c^)	0.90	.002	0.35 to 1.46
Age	–0.07	.01	–0.12 to –0.01
Alcohol user (yes vs no)	–0.60	.04	–1.16 to –0.04
Drug user (yes vs no)	–1.45	.16	–3.45 to 0.56
Current smoker (yes vs no)	–0.74	.06	–1.51 to 0.04
Income (≥CNY 5000/US $743.89^d^ vs below)	0.27	.37	–0.32 to 0.86
Have a steady partner (vs no)	1.10	<.001	0.57 to 1.64

^a^QOL: quality of life.

^b^CHW: cisgender heterosexual women.

^c^SGMW: sexual and gender minority women.

^d^CNY 1=US $0.15.

**Table 8:** Regressions of sexual identity on the environment QOL^a^.

Characteristics	Environment QOL (R-square=4.4%)		
	Coefficient	*P* value	95% CI
Sexual identity (CHW^b^ vs SGMW^c^)	0.67	.008	0.17 to 1.17
Age	–0.03	.20	–0.08 to 0.02
Alcohol user (yes vs no)	0.01	.97	–0.50 to 0.51
Drug user (yes vs no)	–0.21	.82	–2.01 to 1.59
Current smoker (yes vs no)	–0.74	.04	–1.44 to –0.05
Income (≥CNY 5000/US $743.89^d^ vs below)	0.41	.13	–0.12 to 0.94
Have a steady partner (vs no)	0.30	.23	–0.19 to 0.78

^a^QOL: quality of life.

^b^CHW: cisgender heterosexual women.

^c^SGMW: sexual and gender minority women.

^d^CNY 1=US $0.15.

**Table 9:** Regressions of sexual identity on the overall QOL^a^.

Characteristics	Overall QOL (R-square=10.3%)		
	Coefficient	*P* value	95% CI
Sexual identity (CHW^b^ vs SGMW^c^)	3.47	<.001	1.68 to 5.26
Age	–0.08	.37	–0.24 to 0.09
Alcohol user (yes vs no)	–1.10	.23	–2.91 to 0.71
Drug user (yes vs no)	–0.02	.99	–6.47 to 6.43
Current smoker (yes vs no)	–3.70	.004	–6.19 to –1.21
Income (≥CNY 5000/US $743.89^d^ vs below)	1.64	.09	–0.26 to 3.54
Have a steady partner (vs no)	2.45	.006	0.72 to 4.18

^a^QOL: quality of life.

^b^CHW: cisgender heterosexual women.

^c^SGMW: sexual and gender minority women.

^d^CNY 1=US $0.15.

**Table 10:** Regressions of sexual identity on mental health (depression).

Characteristics	Depression (R-square=8.5%)		
	Coefficient	*P* value	95% CI
Sexual identity (CHW^a^ vs SGMW^b^)	-1.74	.002	–2.82 to –0.66
Age	-0.07	.16	–0.17 to 0.03
Alcohol user (yes vs no)	0.86	.12	–0.24 to 1.95
Drug user (yes vs no)	-2.01	.31	–5.91 to 1.89
Current smoker (yes vs no)	2.50	.001	0.99 to 4.00
Income (≥CNY 5000/US $743.89^c^ vs below)	-0.03	.95	–1.18 to 1.12
Have a steady partner (vs no)	-0.42	.43	–1.47 to 0.63

^a^CHW: cisgender heterosexual women.

^b^SGMW: sexual and gender minority women.

^c^CNY 1=US $0.15.

**Table 11:** Regressions of sexual identity on mental health (anxiety).

Characteristics	Anxiety (R-square=6.9%)		
	Coefficient	*P* value	95% CI
Sexual identity (CHW^a^ vs SGMW^b^)	-1.11	.02	–2.07 to –0.15
Age	-0.03	.46	–0.12 to 0.06
Alcohol user (yes vs no)	1.06	.03	0.09 to 2.04
Drug user (yes vs no)	-2.34	.19	–5.81 to 1.13
Current smoker (yes vs no)	1.87	.006	0.53 to 3.21
Income (≥CNY 5000/US $743.89^c^ vs below)	-0.33	.53	–1.35 to 0.70
Have a steady partner (vs no)	-0.68	.15	–1.61 to 0.25

^a^CHW: cisgender heterosexual women.

^b^SGMW: sexual and gender minority women.

^c^CNY 1=US $0.15.

**Table 12:** Regressions of sexual identity on mental health (self-esteem).

Characteristics	Self-esteem (R-square=7.8%)		
	Coefficient	*P* value	95% CI
Sexual identity (CHW^a^ vs SGMW^b^)	1.01	.02	0.15 to 1.86
Age	0.00	.97	–0.08 to 0.08
Alcohol user (yes vs no)	0.04	.92	–0.82 to 0.91
Drug user (yes vs no)	1.58	.31	–1.50 to 4.66
Current smoker (yes vs no)	-1.91	.002	–3.10 to –0.72
Income (≥CNY 5000/US $743.89^c^ vs below)	1.33	.004	0.42 to 2.23
Have a steady partner (vs no)	0.60	.16	–0.23 to 1.43

^a^CHW: cisgender heterosexual women.

^b^SGMW: sexual and gender minority women.

^c^CNY 1=US $0.15.

### Mediation Effects Between Sexual Identity and the QOL via Mental Health

**Figure 2:**
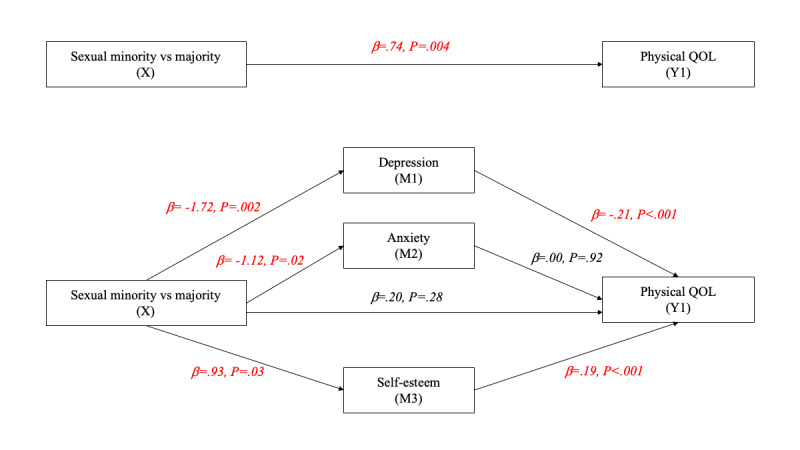
Parallel mediation model for the physical QOL. M: mediator; QOL: quality of life.

**Figure 3:**
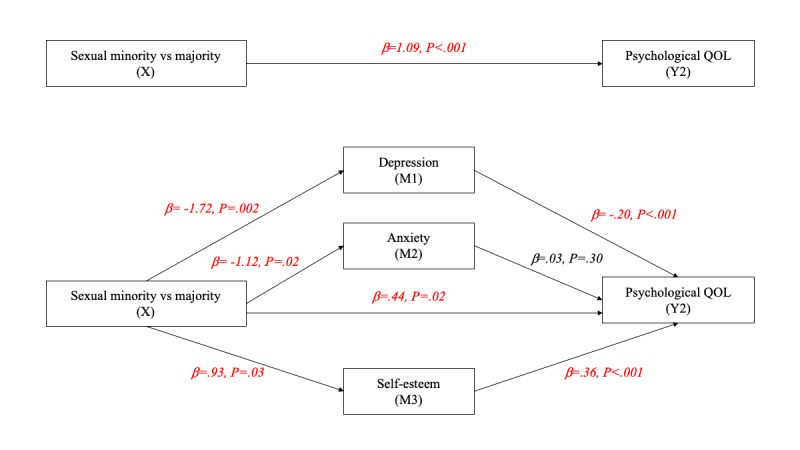
Parallel mediation model for the psychological QOL. M: mediator; QOL: quality of life.

**Figure 4:**
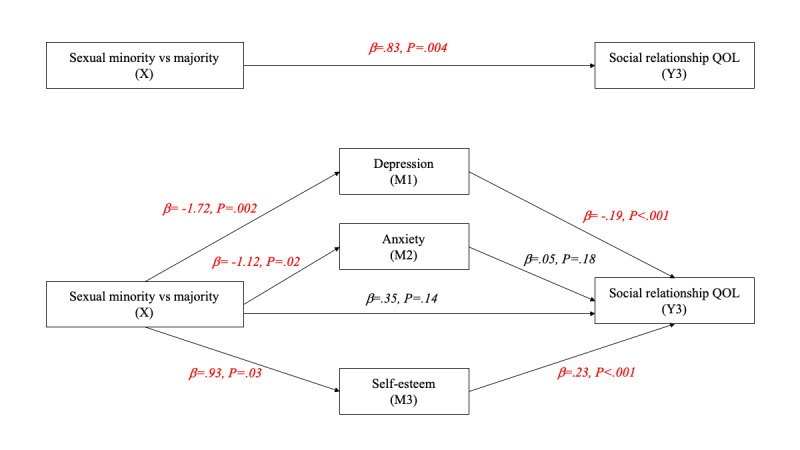
Parallel mediation model for the social relationship QOL. M: mediator; QOL: quality of life.

**Figure 5:**
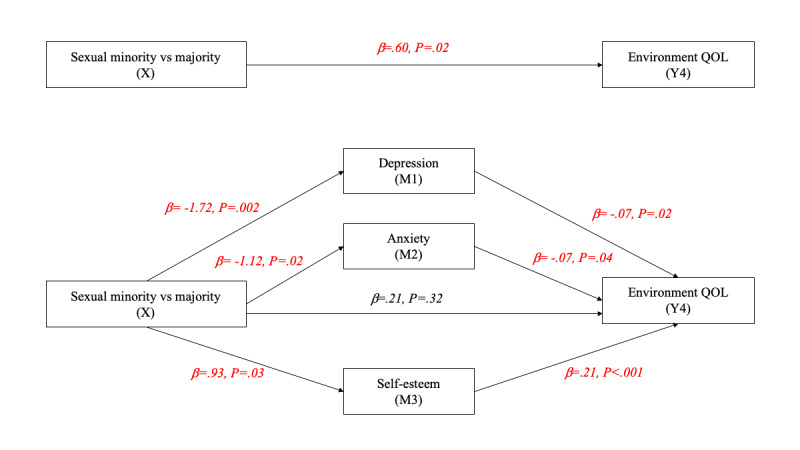
Parallel mediation model for the environment QOL. M: mediator; QOL: quality of life.

**Figure 6:**
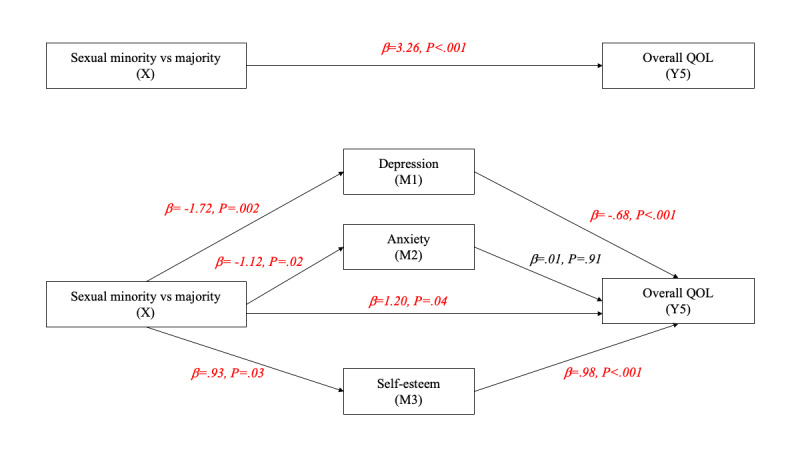
Parallel mediation model on the overall QOL. M: mediator; QOL: quality of life.

Figures 2-6 and Table 13 show the effects of parallel mediation analyses and the corresponding unstandardized effect coefficients of mental health on the relationship between sexual identity status and the QOL. As demonstrated, individuals’ sexual identity was significantly associated with depression (β=–1.72, *P*=.002), anxiety (β=–1.12, *P*=.02), and self-esteem (β=.93, *P*=.03). As mediators, depression and self-esteem were significantly associated with the QOL in all domains as well as the overall QOL (β ranging from –.07 to –.68 and .19 to .98, respectively; all *P*<.05), while anxiety was only significantly associated with the QOL in the environment domain (β=–.07, *P*=.04). Likewise, the indirect effects of depression, anxiety, and self-esteem were only significant (95% CI did not contain 0) in the environment domain of the QOL, while the indirect effects of depression and self-esteem were significant in all other domains of the QOL as well as the overall QOL, indicating that anxiety is only a mediator in the environment domain but not in other aspects. After considering the mediation effects of depression, anxiety, and self-esteem, the direct effects (c’) between sexual identity and the physical QOL (β=.20, *P*=.28), social relationship QOL (β=.35, *P*=.14), and environment QOL (β=.21, *P*=.32) were no longer statistically signiﬁcant, supporting complete mediation effects. In contrast, the direct effects between sexual identity and the psychological QOL (β=.44, *P*=.02) and the overall QOL (β=1.20, *P*=.04) remained statistically signiﬁcant, supporting partial mediation effects.

**Table 13:** Mediation effects of mental health on the relationship between sexual identity and the QOL^a^.

Dependent variable (Y)		Total effect (c)				Direct effect (c’)				X→M^b^ (a1, a2, a3)^c^				M→Y (b1, b2, b3)^d^				Indirect effect (a×b)		
		β^e^	*P* value	SE	β		*P* value	SE	β		*P* value	SE	β		*P* value	SE	β		SE	95% CI
**Sexual identity → physical QOL (X→Y1)**																				
	X→M1→Y1	.74	.004	.25	.20		.28	.18	–1.72		.002	.55	–.21		<.001	.03	.37		.13	0.12 to 0.65
	X→M2→Y1	—^f^	—	—	—		—	—	–1.12		.02	.49	.00		.92	.03	.00		.04	–0.08 to 0.08
	X→M3→Y1	—	—	—	—		—	—	.93		.03	.43	.19		<.001	.02	.17		.08	0.02 to 0.35
**Sexual identity → psychological QOL (X→Y2)**																				
	X→M1→Y2	1.09	<.001	.29	.44		.02	.18	–1.72		.002	.55	–.20		<.001	.03	.35		.13	0.11 to 0.61
	X→M2→Y2	—	—	—	—		—	—	–1.12		.02	.49	.03		.30	.03	–.03		.04	–0.12 to 0.03
	X→M3→Y2	—	—	—	—		—	—	.93		.03	.43	.36		<.001	.02	.33		.15	0.03 to 0.63
**Sexual identity → social relationship QOL (X→Y3)**																				
	X→M1→Y3	.83	.004	.28	.35		.14	.24	–1.72		.002	.55	–.19		<.001	.03	.33		.12	0.11 to 0.59
	X→M2→Y3	—	—	—	—		—	—	–1.12		.02	.49	.05		.18	.04	–.05		.05	–0.18 to 0.03
	X→M3→Y3	—	—	—	—		—	—	.93		.03	.43	.23		<.001	.03	.21		.10	0.02 to 0.41
**Sexual identity → environment QOL (X→Y4)**																				
	X→M1→Y4	.60	.02	.25	.21		.32	.22	–1.72		.002	.55	–.07		.02	.03	.12		.07	0.01 to 0.28
	X→M2→Y4	—	—	—	—		—	—	–1.12		.02	.49	–.07		.04	.03	.08		.06	0.00 to 0.20
	X→M3→Y4	—	—	—	—		—	—	.93		.03	.43	.21		<.001	.03	.19		.09	0.02 to 0.37
**Sexual identity → overall QOL (X→Y5)**																				
	X→M1→Y5	3.26	<.001	.91	1.20		.04	.59	–1.72		.002	.55	–.68		<.001	.09	1.17		.43	0.37 to 2.06
	X→M2→Y5	—	—	—	—		—	—	–1.12		.02	.49	.01		.91	.09	–.01		.11	–0.26 to 0.21
	X→M3→Y5	—	—	—	—		—	—	.93		.03	.43	.98		<.001	.07	.91		.42	0.09 to 1.75

^a^QOL: quality of life.

^b^M: mediator.

^c^a1, a2, and a3 are the effects of the sexual identity (X) on the mental health mediators depression (M1), anxiety (M2), and self-esteem (M3), respectively.

^d^Correspondingly, b1, b2, and b3 are the effects of M1, M2, and M3, respectively, on the QOL (Y).

^e^Unstandardized coefficients (β) are reported. Bootstrap sample size=5000.

^f^Not applicable.

## Discussion

### Principal Findings

The QOL is widely used to describe individuals’ perceptions of general and holistic health [27,52]. SGMW in this study reported significantly lower levels of overall and domain-specific QOL, higher levels of depression and anxiety, and lower levels of self-esteem compared to CHW. Our ﬁndings are consistent with several large-scale reviews of the mental health of sexual minority populations in Western countries [3,10], all suggesting that people belonging to sexual minorities are at higher risk of mental health disorders. Meanwhile, our study findings are consistent with the findings in Hong Kong, both showing that Chinese sexual minority populations report poorer levels of the QOL than CH people [28]. In terms of positive mental health, the CHW in our study reported significantly higher levels of self-esteem than the SGMW, which may be related to the fact that the CH identity is mainstreamed and encouraged in Chinese society, suggesting the need to consider specific social contexts when exploring the health of SGM populations.

In terms of cultural issues affecting the QOL and mental health of SGMW in China, it is critical to consider the deep-rooted traditional Confucian Chinese culture that highly values family and inheritance. Specifically, being a member of SGM populations in China is associated with internalized homophobia and stressful feelings in the face of social expectations [25,59,60], which is found to be negatively correlated with lower self-evaluation [61] and social well-being [62]. Compared to the twentieth century, the living conditions of sexual minority populations in China do not seem to have changed much and lesbians have always been more hidden and invisible than gays [63]. According to a recent national survey on sexuality in China, for women born in the 1980s and 1990s (similar to the age group of the population surveyed in this study), 30.3% and 18.1%, respectively, believe homosexual sex is always wrong, and both rates are higher among men in the same age groups [64].

Furthermore, despite a similar cultural background, the overall participants in our study (mainly from mainland China) scored lower than SGMW in Taiwan using the same RSES [25], which may be related to the differences in policy—same-sex marriage has been legalized in Taiwan since 2019 [65]. However, governments in mainland China, Hong Kong, and Macau do not recognize same-sex marriage, which may further lead the sexual minority populations to feel isolated and fear social condemnation [60]. Despite the fact that homosexuality is no longer classified as a pathology under the *Chinese Classification of Mental Disorders* since 2001 [66], the SGMW in our study reported a more worrisome QOL and mental health disorders than the CHW. Thus, future researchers should not only pay attention to SGM populations’ current health status but also fully consider social contexts and living environments before designing adaptive health improvement interventions.

Both correlation and regression analyses in this study showed that the overall QOL as well as the QOL in each domain are significantly associated with depression, anxiety, and self-esteem, which is in line with previous studies. Specifically, depression was shown to negatively impact the QOL in our study, as has been seen in similar pathway analysis studies in other populations [67]. Previous studies have also suggested that people with anxiety report an impaired QOL compared to the general population [68], and both depression and anxiety play an important role in the QOL [69], supporting our study results. On the hypothesized mediation effects of mental health, our results indicated that sexual identity not only directly affects the QOL but also indirectly impacts the QOL through the complete or partial mediating effects of mental health variables, which is consistent with previous studies supporting the mediation effects of mental health [56,70]. The pathways from sexual identity to a reduced QOL shown in our study can be attributed to depression, anxiety, and self-esteem. SGMW reported higher levels of depression and anxiety but lower levels of self-esteem than CHW, which was subsequently related to lower levels of the QOL. To minimize the negative effects of sexual identity on the QOL, mental health may be promoted through interventions targeting SGMW.

Given the prior neglect of the health status of SGM populations and the worrisome levels of the QOL and mental health among SGMW, the participants in this study, our results also have significant implications for professional practice. It is recommended that health and social care professionals proactively attend to the mental health of people belonging to sexual minorities when designing or providing interventions. Notably, we found that the proportions of current smokers and alcohol users were significantly higher in SGMW than in CHW, which is supported by previous studies [71-74]. As shown in the multiple linear regressions, current smokers had poorer levels of the QOL in each domain as well as the overall QOL, which is also consistent with the review conclusions that smoking is negatively associated with the QOL [75]. In addition, the current smoking status was significantly associated with all mental health outcomes in this study, as indicated by higher levels of depression and anxiety and lower levels of self-esteem in current smokers compared to nonsmokers, so the substance use among SGMW in China requires more attention and corresponding improvement programs are needed. Existing evidence shows that smoking cessation is associated with reduced depression and anxiety and could significantly improve the QOL as well [76]. With regard to specific smoking cessation programs for SGM populations, a review summarized that interventions targeting SGM populations are more effective than those targeting the general population [74] and a connection to specific sexual minority communities could protect young sexual minority women in the United States from the risk of smoking [71]; therefore, further research to examine whether sexual minority community engagement can reduce the likelihood of smoking among Chinese SGM populations is warranted.

Although there were no significant associations between having a steady partner and mental health variables in our study, being in a steady relationship was found to be significantly positively associated with the overall QOL, psychological QOL, and social relationship QOL. Such findings that the relationship status could positively affect the QOL are consistent with a published review showing that being in a relationship with a regular partner is a protective factor for health [77], so future interventions that take relationship factors into account may be promising. There is also ample evidence that social support has protective effects on the mental health and QOL of sexual minority populations [9,29,78,79], and perceived support from participants’ partners is related to lower risk of depression among SGMW [25]. In contrast, stressors, such as victimization and lower social or family support, may contribute to higher levels of depression among sexual minority populations compared to CH individuals [30]. However, there are few studies examining the comprehensive factors influencing the holistic health of Chinese SGM individuals, and studies only on Chinese SGMW are basically nonexistent; thus, it is strongly recommended that future researchers conduct specific explorations targeting female minority populations.

Sexual minority populations have been found to experience higher rates of physical and mental health disorders and poorer levels of the QOL [2-9,80]; thus, interventions should be designed to help improve their holistic health, especially the QOL. The ﬁnding of this study that mental health could mediate the association between sexual identity and the QOL highlights the need for all relevant stakeholders to assess SGM populations’ mental health when providing health care or social service. Based on our findings, we also recommend that future interventions aimed at improving the QOL of sexual and gender minorities also emphasize positive mental health components, such as self-esteem, thereby improving their QOL.

To sum up, this is the ﬁrst study to examine both the QOL and mental health among Chinese adult women with diverse sexual identities and also the first to conduct comparisons between SGMW and CHW, showing that SGMW experience worse levels of the QOL and mental health compared to CHW. Although the associations between the QOL and mental health are well established in general populations, there has been little research on these relationships in people of different gender and sexual orientation identities, so this study is also the first to report the interrelationships between sexual identity and the QOL as well as mental health (depression, anxiety, and self-esteem). Moreover, our study is also the first to explore and confirm the mediating role of mental health in the impact of sexual identity on the QOL.

### Limitations

This study had several limitations. First, although the QOL and mental health variables explored in this study are generally investigated as separate constructs, this has certain limitations as the meanings of these concepts are intertwined and may influence each other. Although due to the cross-sectional design of this study, the comprehensive relationships within these concepts could not be adequately explored, further studies using more diverse research methodologies, such as qualitative approaches or longitudinal designs, are needed. Second, most of our study participants were young, and nearly half had no paid employment or were still full-time students; therefore, the results might not be generalizable to the broader Chinese female population. Third, although the sample size was sufficient for comparisons between SGMW as a whole and CHW, subgroup analyses by specific sexual orientation may not be powerful enough. Future studies with larger, more diverse, and representative samples are needed.

### Conclusion

This study demonstrated that SGMW have lower levels of the QOL, higher levels of depression and anxiety symptoms, and lower levels of self-esteem than heterosexual women. The overall QOL and QOL in each domain were found to be positively associated with good mental health outcomes, with moderate-to-strong correlations. Multiple linear regressions found that being a sexual or gender minority, current smoker, and woman with no steady partner are associated with a worse overall QOL. Mediation analysis showed that depression, anxiety, and self-esteem play significant mediating roles in the relationship between sexual identity and the QOL in Chinese adult women, affirming the importance of assessing mental health when investigating or improving the QOL. Longitudinal studies and evidence-based intervention programs are further needed, with a particular focus on sexual minority women.

## Appendix

Multimedia Appendix 1 Comparisons of quality of life and mental health among 5 sexual orientation groups.

## Abbreviations

CH: cisgender heterosexual
CHW: cisgender heterosexual women
GAD-7: 7-item Generalized Anxiety Disorder scale
PHQ-9: 9-item Patient Health Questionnaire
QOL: quality of life
RSES: Rosenberg Self-Esteem Scale
SGM: sexual and gender minority
SGMW: sexual and gender minority women
WHOQOL-BREF: World Health Organization Quality of Life–abbreviated short version
