# Supplementary material for: Quality of Life and Mental Health of Chinese Sexual and Gender Minority Women and Cisgender Heterosexual Women: Cross-sectional Survey and Mediation Analysis
Source: JMIR Public Health Surveill. 2023 Feb 22;9:e42203. doi: 10.2196/42203 (PMC9996424; doi:10.2196/42203)
Supplement: Multimedia Appendix 1 [file publichealth_v9i1e42203_app1.docx]

# Table S1 Comparisons of quality of life and mental health among five sexual orientation groups

|  |  | Homosexual (1) | Heterosexual (2) | Bisexual (3) | Pansexual (4) | All others (5) | *P* | Bonferroni comparisons |
| --- | --- | --- | --- | --- | --- | --- | --- | --- |
|  |  | (n=71) | (n=250) | (n=88) | (n=52) | (n=48) |  |  |
| **Quality of life (WHOQOL-BREF)** | | |  |  |  |  |  |  |
|  | Physical | 14.38(2.73) | 14.63(2.39) | 13.76(2.78) | 12.59(3.18) | 12.67(2.44) | <.001 | 1>4^**^;1>5^**^;2>4^**^;2>5^**^ |
|  | Psychological | 13.68(3.31) | 14.57(2.65) | 13.39(3.14) | 12.45(3.64) | 11.54(3.01) | <.001 | 1>5^**^;2>3^*^;2>4^**^;2>5^**^;3>5^**^ |
|  | Social relationship | 13.39(3.11) | 14.23(2.61) | 13.32(3.51) | 12.62(3.90) | 12.39(2.62) | <.001 | 2>4^**^;2>5^**^ |
|  | Environment | 13.94(2.86) | 14.18(2.35) | 13.57(2.82) | 13.38(3.12) | 11.85(2.42) | <.001 | 1<2^**^;2>5^**^;3>5^**^;4>5^*^ |
|  | Total score | 55.40(10.33) | 57.61(8.34) | 54.04(10.41) | 51.03(12.00) | 48.45(8.67) | <.001 | 1>5^**^;2>3^*^;2>4^**^;2>5^**^;3>5^*^ |
| **Mental health** | |  |  |  |  |  |  |  |
|  | Depression (PHQ-9) | 7.49(5.92) | 6.50(5.11) | 8.98(5.70) | 10.79(7.43) | 10.19(6.43) | <.001 | 1<4^*^;2<3^**^;2<4^**^;2<5^**^ |
|  | Anxiety (GAD-7) | 5.69(5.62) | 5.04(4.77) | 7.03(5.12) | 7.81(6.01) | 7.63(5.21) | <.001 | 2<3^*^;2<4^**^;2<5^*^ |
|  | Self-esteem (RSES) | 28.93(4.91) | 29.72(4.18) | 28.52(4.69) | 27.02(5.54) | 26.96(4.51) | <.001 | 2>4^**^;2>5^**^ |

Note: SD (standard deviation); ^**^ (*P*<.01); ^*^ (*P*<.05).

WHOQOL-BREF (World Health Organization Quality of Life-abbreviated short version); PHQ-9 (The 9-item Patient Health Questionnaire); GAD-7 (The 7-item Generalized Anxiety Disorder scale); RSES (The Rosenberg Self-Esteem Scale).

If there was significant difference, then Bonferroni comparisons were conducted.
